# Stress increases intracardiac 4D flow cardiovascular magnetic resonance -derived energetics and vorticity and relates to VO_2_max in Fontan patients

**DOI:** 10.1186/s12968-019-0553-4

**Published:** 2019-07-25

**Authors:** Vivian P. Kamphuis, Mohammed S. M. Elbaz, Pieter J. van den Boogaard, Lucia J. M. Kroft, Hildo J. Lamb, Mark G. Hazekamp, Monique R. M. Jongbloed, Nico A. Blom, Willem A. Helbing, Arno A. W. Roest, Jos J. M. Westenberg

**Affiliations:** 1 Department of Pediatrics division of Pediatric Cardiology, Leiden University Medical Center, Leiden, the Netherlands; 2 Netherlands Heart Institute, Utrecht, The Netherlands; 3 Department of Radiology Feinberg School of Medicine, Northwestern University, Chicago, USA; 4 Department of Radiology, Leiden University Medical Center, Leiden, the Netherlands; 5 Department of Cardiothoracic Surgery, Leiden University Medical Center, Leiden, the Netherlands; 6 Department of Cardiology, Leiden University Medical Center, Leiden, the Netherlands; 7 Department of Pediatrics division of Pediatric Cardiology, Academic Medical Center, Amsterdam, the Netherlands; 8 Department of Pediatrics, division of Pediatric Cardiology, Erasmus Medical Center, Rotterdam, the Netherlands; 9 Department of Pediatrics division of Pediatric Cardiology, Radboud university Medical Center, Nijmegen, the Netherlands

**Keywords:** 4D flow CMR, Flow, Kinetic energy: energy loss, Vorticity, Fontan

## Abstract

**Background:**

We hypothesize that dobutamine-induced stress impacts intracardiac hemodynamic parameters and that this may be linked to decreased exercise capacity in Fontan patients**.** Therefore, the purpose of this study was to assess the effect of pharmacologic stress on intraventricular kinetic energy (KE), viscous energy loss (EL) and vorticity from four-dimensional (4D) Flow cardiovascular magnetic resonance (CMR) imaging in Fontan patients and to study the association between stress response and exercise capacity.

**Methods:**

Ten Fontan patients underwent whole-heart 4D flow CMR before and during 7.5 μg/kg/min dobutamine infusion and cardiopulmonary exercise testing (CPET) on the same day. Average ventricular KE, EL and vorticity were computed over systole, diastole and the total cardiac cycle (vorticity_vol_avg cycle_, KE_avg cycle,_ EL_avg cycle_). The relation to maximum oxygen uptake (VO_2_ max) from CPET was tested by Pearson’s correlation or Spearman’s rank correlation in case of non-normality of the data.

**Results:**

Dobutamine stress caused a significant 88 ± 52% increase in KE (KE_avg cycle_: 1.8 ± 0.5 vs 3.3 ± 0.9 mJ, *P* < 0.001), a significant 108 ± 49% increase in EL (EL_avg cycle_: 0.9 ± 0.4 vs 1.9 ± 0.9 mW, *P* < 0.001) and a significant 27 ± 19% increase in vorticity (vorticity_vol_avg cycle_: 3441 ± 899 vs 4394 ± 1322 mL/s, *P* = 0.002). All rest-stress differences (%) were negatively correlated to VO_2_ max (KE_avg cycle_: *r* = − 0.83, *P* = 0.003; EL_avg cycle_: *r* = − 0.80, *P* = 0.006; vorticity_vol_avg cycle_: *r* = − 0.64, *P* = 0.047).

**Conclusions:**

4D flow CMR-derived intraventricular kinetic energy, viscous energy loss and vorticity in Fontan patients increase during pharmacologic stress and show a negative correlation with exercise capacity measured by VO_2_ max.

## Background

The Fontan procedure is a palliative surgical procedure for patients with complex congenital intracardiac deformations in whom a biventricular circulation cannot be created [1]. Survival of patients after the procedure has increased drastically over the past decades [2, 3], but still, these patients exhibit diminished exercise capacity, which is related to a worse functional health status [4–6]. Several factors have been identified that play a role in the reduced exercise capacity in these patients, such as an inadequate ventricular response to exercise, reduced pulmonary and pulmonary vascular functions, muscle weakness and energy loss in the Fontan tunnel [7–12]. Four-dimensional (4D) flow cardiovascular magnetic resonance (CMR) imaging enables comprehensive assessment of in vivo blood flow patterns and quantification of hemodynamic parameters from blood flow velocity such as kinetic energy (KE), viscous energy loss (EL, the KE that is lost due to viscosity-induced frictional forces) and vorticity (a measure of the local spinning of blood particles) [13]. Previous studies have suggested that such vortical spinning patterns of blood flow (vortex formation) may help preserve kinetic energy by minimizing energy losses, hence enabling energy-efficient blood transportation [14, 15].

Recently, altered intracardiac flow patterns, levels of kinetic energy and vortex formation in the intraventricular blood flow pattern were observed in Fontan patients [16–18]. Furthermore, reduced exercise capacity was linked to EL in the total cavopulmonary connection (TCPC) of Fontan patients [10, 12]. However, currently the link between altered intracardiac hemodynamics during stress and exercise capacity remains unknown. We hypothesize that dobutamine-induced stress would impact these intracardiac hemodynamic parameters and that this may be linked to decreased exercise capacity in Fontan patients.

Therefore, the purpose of this study was 1) to non-invasively assess the influence of pharmacologic stress on KE, EL and vorticity measured by 4D flow CMR in intraventricular blood flow in Fontan patients and 2) to study the association between the hemodynamic stress response in terms of KE, EL and vorticity and exercise capacity assessed by maximum oxygen uptake (VO_2_ max) from cardiopulmonary exercise tests (CPET).

## Methods

### Study population

Twenty-six Fontan patients were prospectively evaluated in this study at the Leiden University Medical Center (LUMC) as part of a multicenter study that was approved by the Medical Ethical Committee of the Erasmus Medical Center in Rotterdam (MEC-2014-326, NL48188.078.14), with local approval of the Medical Ethical Committee of the LUMC, The Netherlands. Informed consent was obtained from all of these participants. All methods were performed in accordance with the relevant guidelines and regulations. All patients underwent CPET and a CMR on the same day. Patients were included in the present study when the dobutamine stress CMR scan with 4D flow CMR at rest and stress was fully completed: which was the case in 10/26 (38%) patients. Reasons for not finishing the complete scan were: 10 (38%) patients refused stress testing, in 4 (15%) patients stress testing could only be completed without 4D flow CMR and in 2 (8%) patients dobutamine infusion had to be stopped because of ventricular extra systoles.

### Cardiopulmonary exercising testing

Cardiopulmonary exercising testing was performed on an upright bicycle ergometer (General Electric Healthcare, Waukesha, Wisconsin, USA). Starting wattage and workload increase per minute were based on patient’s baseline condition and defined by the attending physician. Patients were encouraged to exercise until exhaustion. VO_2_ max was derived from the CPET following previously published methods [19].

### Cardiovascular magnetic resonance acquisition and image analysis

A complete CMR scan including whole-heart 4D flow CMR was obtained on a 3 T scanner (Ingenia, Philips Healthcare, Best, The Netherlands) during rest and with dobutamine 7.5 μg/kg/min. Details on CMR acquisition are provided in Appendix 1. Retrospective gating was used with 30 phases reconstructed to represent one cardiac cycle. Free breathing was allowed without using motion suppression such as gating by navigator or bellow, but we acquired three signal averages to minimize effects of breathing motion. Dobutamine infusion was reduced to 5 μg/kg/min or stopped in case of a > 50% increase in heart rate (HR), systolic, or diastolic blood pressure or when significant rhythm disorders occurred. The test was stopped when the patient experienced discomfort. Contour segmentation was performed using in-house developed *MASS* software (Medis Medical Imaging Systems, Leiden, The Netherlands) by one observer (VPK) with over 3 years of experience in CMR and verified by a radiologist (LJMK) with over 20 years of experience in CMR. Ventricular volume was segmented in all slices and phases in the cine transversal images (papillary muscles were included in the ventricular volume; remaining parts of the septum in “biventricular” patients were not included in the ventricular volume). Segmented volumes were then used to compute stroke volume (SV) as: end-diastolic volume − end-systolic volume. Cardiac output (CO) was computed as *SV* × *HR*. We used the sphericity index to measure the change in the shape of the ventricle between rest and stress. The sphericity index was calculated as the short to long axis ratio: ventricular width/ventricular height at end-systole and end-diastole in the 2D cine transversal images. Time points of systolic and diastolic phases were derived from the flow-time curves that resulted from retrospective valve tracking assessing the inflow and outflow of the systemic ventricle in Fontan patients, following previously published methods [20]. Segmentation of the ventricular cavity in the 4D Flow CMR acquisition, that is required for the energy and vorticity analysis, was obtained by transforming the available time-varying segmentation of multi-slice cine transversal anatomical acquisition to the 4D Flow CMR data [21]. This procedure was performed using in-house developed *MASS* software by one observer (VPK) with over 3 years of experience in CMR and verified by researcher (MSME) with 6 years of experience in CMR. Aliasing of 4D Flow velocity images was visually checked in the source images (all three velocity components) and unwrapped using a sliding velocity scaling in *MASS* software. Following previous work [18, 21–23], to account for potential patient-motion related misalignment between the 4D flow CMR and transversal anatomical cine acquisitions, automated image-based 3D rigid registration by mutual information was performed using the phase with the maximal depiction of the ventricular cavity in both scans with the Elastix image registration toolbox [24].

### Computation of KE, EL and vorticity in the 4D flow CMR data

The amount of intraventricular KE was computed as ½ *mv*^*2*^, with *m* as the mass representing the voxel volume multiplied by the density of blood (1.025 g/mL) and *v* as the magnitude of the 3-directional velocity vector acquired from 4D flow CMR. For each acquired time-phase, volumetric KE was then computed by integrating (by cumulative sum) the computed KE over the segmented 3D ventricular volume. The KE was quantified by the time-average kinetic energy over systole (KE_avg systole_), diastole (KE_avg diastole_) and the complete cardiac cycle (KE_avg cycle_), all expressed in milliJoule (mJ) [23]. EL was computed from 4D flow CMR using the dissipation terms from the Navier-Stokes energy equations, following recently published method [21, 23], assuming blood as a Newtonian fluid. Average EL in milliWatt (mW) was computed over systole (EL_avg systole_), diastole (EL_avg diastole_) and the complete cardiac cycle (EL_avg cycle_).

The vorticity metric (mathematically computed as the curl of velocity field – here derived from 4D flow CMR) enables direct quantification of the strength of vortical flow at every voxel (i.e. voxel-wise vorticity) as well as the integrating (total sum) of the total vorticity over the entire chamber volume (i.e. indicated here as vorticity_vol). More technical details can be found in: [25]. Voxel-wise vorticity magnitude (in 1/s) was computed for each acquired time-phase and then integrated over the total ventricular volume [23]. To differentiate from voxel-wise vorticity, we will refer to this integrated volumetric vorticity as vorticity_vol (in mL/s). This vorticity_vol is a scalar quantity and does not take the vorticity direction into account. In order to quantify the integral vorticity_vol, the time-averaged vorticity over systole and diastole and the total cardiac cycle (vorticity_vol_avg systole_, vorticity_vol_avg diastole_, vorticity_vol_avg cycle_ respectively) were assessed. All EL, KE and vorticity parameters at rest and stress are also given normalized by SV.

### Statistical analysis

Data analysis was performed using SPSS Statistics (version 23.0 Statistical Package for the Social Sciences (SSPS), International Business Machines, Inc., Armonk, New York, USA). Variables were tested for normal distribution using the Shapiro-Wilk test. Continuous data is reported as mean ± standard deviation (SD) or median [interquartile range (IQR)], in case of non-normality of the data. The absolute differences between rest and stress measurements (stress measurement – rest measurement) were assessed and significance was tested with the paired samples t-test or the Wilcoxon signed-rank test in case of non-normality of the data. Furthermore, the relative differences (as a percentage) between rest and stress measurements [((stress measurement – rest measurement)/rest measurement) × 100] were calculated. Correlation between measurements was tested with the Pearson correlation coefficient or the Spearman’s rank correlation coefficient in case of non-normality of the data. Correlation was classified as follows: > 0.95: excellent; 0.95–0.85: strong; 0.85–0.70: good; 0.70–0.5: moderate; < 0.5: poor. A *P*-value < 0.05 was considered statistically significant.

## Results

Characteristics of the Fontan patients are shown in Table 1. HR increased from 83.4 ± 18.9 bpm to 110.9 ± 20.0 bpm between the rest and dobutamine stress scans (*P* < 0.001). Left ventricular end-diastolic volume and sphericity indices did not change significantly from rest to stress (*P* ≥ 0.12). Stroke volume increased from 80.8 ± 29.4 to 92.0 ± 33.9 mL between the rest and dobutamine stress scans (*P* = 0.004). In one patient, dobutamine infusion was decreased to 5 μg/kg/min because of a > 50% increase in systolic blood pressure from baseline.

**Table 1:** Characteristics of the study group

	Rest	Dobutamine stress	Difference	Difference (%)	*P*-value
	Mean ± SD	Mean ± SD	*Stress-Rest* Mean ± SD or Median [IQR]	*(Stress-rest)/rest* x *100%* Mean ± SD or Median [IQR]	
Characteristics of the study group that underwent the whole protocol (*n* = 10)					
Age (years)	16.5 ± 3.8	n/a	n/a	n/a	n/a
Male (%)	4/10 (40)	n/a	n/a	n/a	n/a
VO_2_ max^a^	30.2 ± 8.6	n/a	n/a	n/a	n/a
Oxygen saturation^a^	93.8 ± 4.4^b^	88.7 ± 4.8^b^	−4.3 ± 3.3	− 5 ± 4	0.008
HR (bpm)	83.4 ± 18.9	110.9 ± 20.0	27.5 ± 8.2	35 ± 12	< 0.001
EDV (mL)	164.4 ± 43.9	156.8 ± 46.0	−7.6 ± 13.9	−5 ± 8	0.12
ESV (mL)	83.6 ± 23.7	64.8 ± 20.9	−21.7 [−25.6 to −14.4]	−26 [−30 to −19]	< 0.001
SV (mL)	80.8 ± 29.4	92.0 ± 33.9	11.1 ± 9.2	14 ± 10	0.004
EF (%)	49.1 ± 8.5	58.6 ± 8.1	9.5 ± 3.2	20 ± 8	< 0.001
CO (L/min)	6.4 ± 1.3	9.7 ± 2.2	3.3 ± 1.3	52 ± 17	< 0.001
Systolic blood pressure (mmHg)	121.2 ± 15.2	150.6 ± 19.3	29.4 ± 12.1	25 ± 11	< 0.001
Diastolic blood pressure (mmHg)	62.7 ± 10.0	70.3 ± 6.0	7.6 ± 9.5	14 ± 19	0.03
Sphericity index systole	1.1 ± 0.3	1.2 ± 0.4	0.1 ± 0.2	2[−8 to 13]	0.44
Sphericity index diastole	1.0 ± 0.1	1.1 ± 0.2	0.05 ± 0.2	2 [−5 tot 9]	0.41

^a^during cardiopulmonary exercise testing

^b^oxygen saturation was missing for one patient

*HR* heart rate, *EDV* end diastolic volume, *ESV* end systolic volume, *SV* stroke volume, *CO* cardiac output, *EF* ejection fraction, *VO2 max* maximal oxygen uptake IQR interquartile range

### Intraventricular KE, EL and vorticity_vol at rest and stress

Table 2 shows the non-normalized results of KE, EL and vorticity_vol at rest and dobutamine stress, for the complete cardiac cycle but also averaged for systole and diastole**.** Average KE significantly increased by 88 ± 52% during the complete cardiac cycle (1.8 ± 0.5 vs 3.3 ± 0.9 mJ, *P* < 0.001). With dobutamine stress, average EL significantly increased by 108 ± 49% when calculated over the complete cardiac cycle (0.9 ± 0.4 vs 1.9 ± 0.9 mW, *P* < 0.001). Average vorticity_vol significantly increased by 27 ± 19% when calculated over the complete cardiac cycle (3441 ± 899 vs 4394 ± 1322 mL/s, *P* = 0.002).

**Table 2:** Quantitative analysis of energetics and vorticity at rest and stress

	Rest	Dobutamine stress	Difference(%)	*P*-value
	Mean ± SD or Median [IQR]	Mean ± SD	*(Stress-rest)/rest x 100%* Mean ± SD or Median [IQR]	
Non-normalized				
KE_avg systole_ (mJ)	2.4 ± 1.1	5.1 ± 1.8	99 [72-195]	< 0.001
KE_avg diastole_ (mJ)	1.4 ± 0.3	2.2 ± 0.7	61 ± 57	0.007
KE_avg cycle_ (mJ)	1.8 ± 0.5	3.3 ± 0.9	88 ± 52	< 0.001
EL_avg systole_ (mW)	1.2 [0.6-1.5]	3.1 ± 1.8	155 ± 61	0.005
EL_avg diastole_ (mW)	0.7 ± 0.2	1.2 ± 0.5	71 ± 66	0.007
EL_avg cycle_ (mW)	0.9 ± 0.4	1.9 ± 0.9	108 ± 49	< 0.001
Vorticity_vol_avg systole_ (mL/s)	3592 ± 1148	4928 ± 1626	37 ± 17	< 0.001
Vorticity_vol_avg diastole_ (mL/s)	3371 ± 770	4086 ± 1216	21 ± 25	0.02
Vorticity_vol_avg cycle_ (mL/s)	3441 ± 899	4394 ± 1322	27 ± 19	0.002
Normalized by stroke volume				
KE_avg cycle_/SV (mJ/mL)	0.023 ± 0.005	0.037 ± 0.009	64 ± 40	< 0.001
EL_avg cycle_/SV (mW/mL)	0.012 ± 0.004	0.021 ± 0.009	81 ± 36	< 0.001
Vorticity_vol_avg cycle_/SV (1/s)	44.1 ± 9.7	49.1 ± 11.1	11 ± 11	0.006

*SD* standard deviation, *KE* kinetic energy, *SV* stroke volume, *EL* viscous energy loss IQR interquartile range

**Fig. 1:**
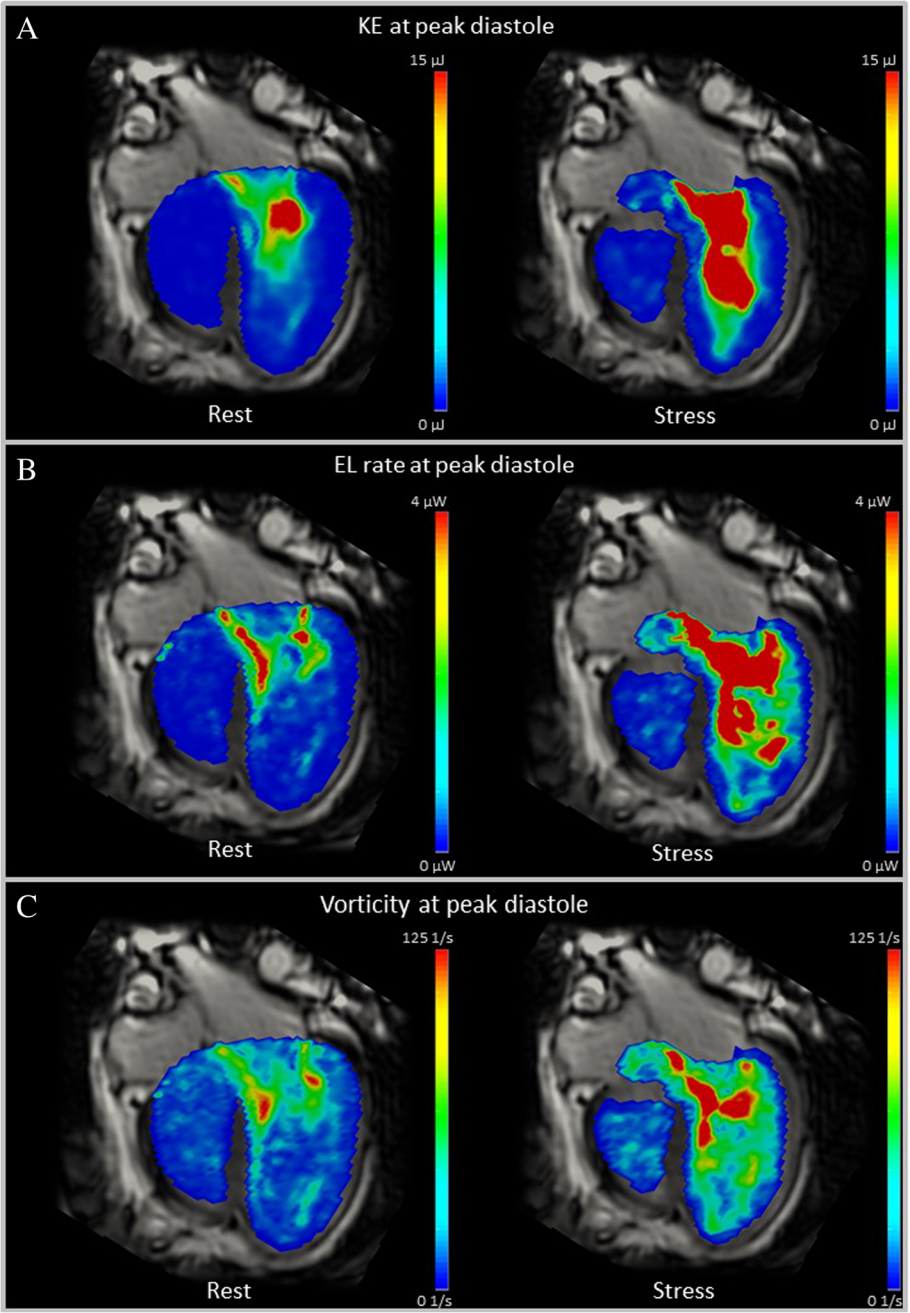
Intracardiac hemodynamics at rest and dobutamine stress in a patient with pulmonary atresia at peak diastolic filling. **a** shows the intraventricular kinetic energy (KE) at rest and stress. **b** shows the intraventricular viscous energy loss (EL) rate at rest and stress. **c** shows the intraventricular vorticity at rest and stress

**Fig. 2:**
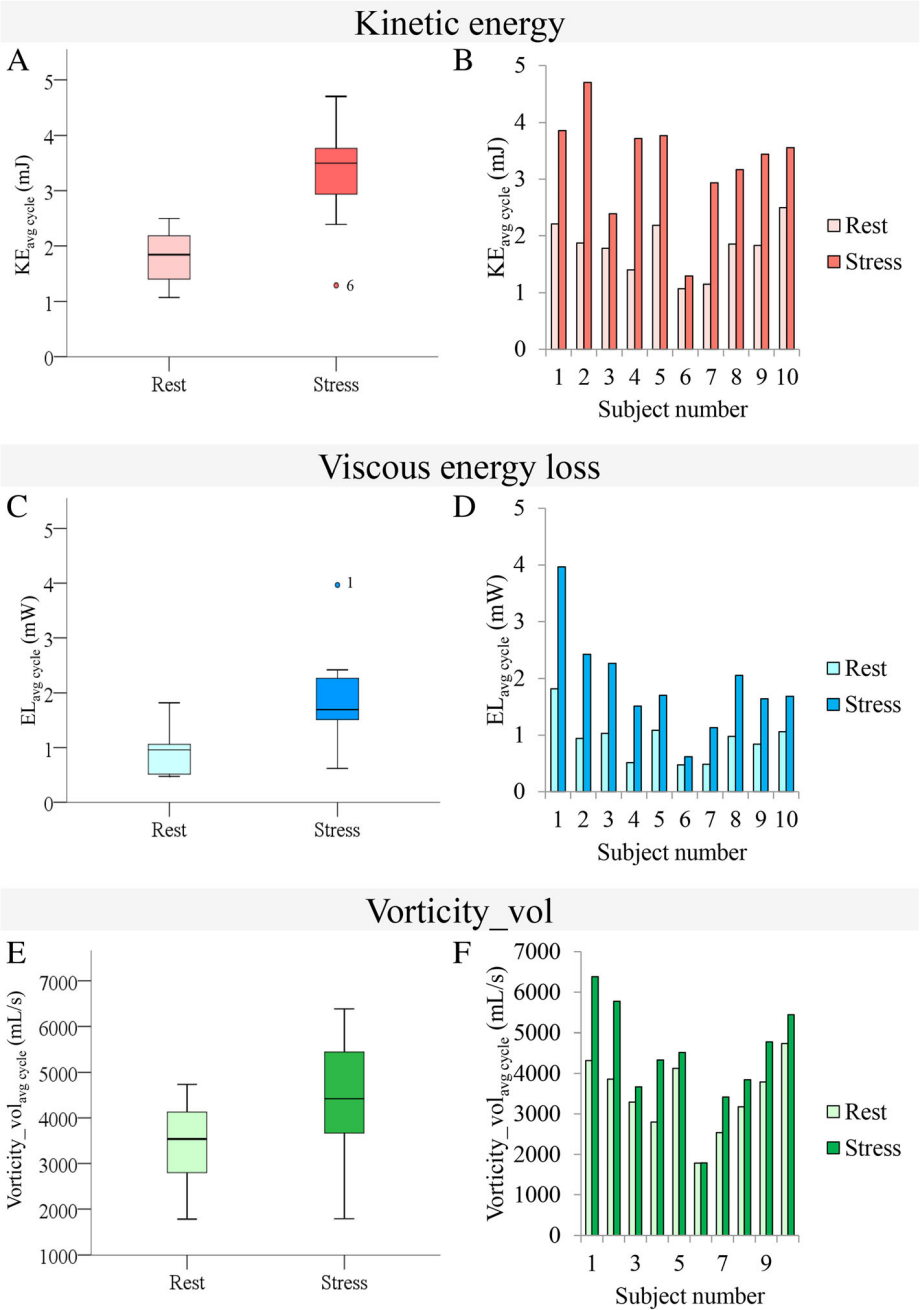
Relative difference in intraventricular kinetic energy (KE), viscous energy loss (EL) and vorticity (vorticity_vol) over the total cardiac cycle between rest and stress. **a** Difference in KE in the total group in rest and stress. There is one outlier: subject 6, who has the lowest KE in stress; **b** Difference in KE per subjects in rest and stress. There is one outlier: subject 1, who has the highest EL in stress; **c** Difference in EL in the total group in rest and stress; **d** Difference in EL per subjects in rest and stress; **e** Difference in vorticity in the total group in rest and stress; **f** Difference in vorticity per subjects in rest and stress. The scales are per voxel –lowest voxel value would be 0 and maximum would be the highest on the corresponding scale

Figure 1 shows an example of the intracardiac hemodynamics during stress in one patient. Boxplots of the absolute differences for the total group and the individual changes between rest and stress values of KE, EL and vorticity_vol are shown in Fig. 2 and Table 3 in Appendix 2. For KE there is one outlier: subject 6, who has the lowest KE in stress. Also for EL there is one outlier: subject 1, who has the highest EL in stress. Results of KE, EL and vorticity_vol at rest and dobutamine stress normalized by SV are also shown in Table 2. Normalized by stroke volume, KE and EL values still showed a significant increase over the total cycle, similar to the non-normalized values.

### Association between difference HR, stroke volume and blood pressure versus KE, EL and vorticity

The difference in HR between rest and stress was not associated with the difference in KE, EL or vorticity_vol (KE_avg cycle_: *r* = − 0.27, *P* = 0.45; EL_avg cycle:_ *r* = 0.01, *P* = 0.97; vorticity_vol_avg cycle_: *r* = − 0.26, *P* = 0.48). The increase in SV (from 2D planimetry) between rest and stress was significantly associated with the difference in vorticity_vol_avg cycle_ (vorticity_vol_avg cycle_: 0.72, *P* = 0.02), but not with the difference in KE or EL *(*KE_avg cycle_: *r* = − 0.46, *P* = 0.19; EL_avg cycle_: *r* = 0.48, *P* = 0.16). There was no correlation between the absolute change in diastolic blood pressure from CMR and the absolute change in diastolic energy parameters (KE: *r* = − 0.11, *P* = 0.77 and EL: *r* = 0.12, *P* = 0.73), or the percentage change in diastolic blood pressure from CMR and percentage change in diastolic energy parameters (KE: *r* = 0.08, *P* = 0.83 and EL: *r* = 0.02, *P* = 0.96). Also, there was no correlation between the absolute change in systolic blood pressure from CMR and absolute change in systolic energy parameters (KE: *r* = − 0.31, *P* = 0.38 and EL: *r* = 0.43, *P* = 0.21). There was a moderate correlation between the percentage change in systolic blood pressure from CMR and the percentage change in systolic KE (*r* = − 0.67, *P* = 0.04), but not in EL (*r* = − 0.35, *P* = 0.32).

### Relation to VO2 max from cardiopulmonary exercise testing

**Fig. 3:**
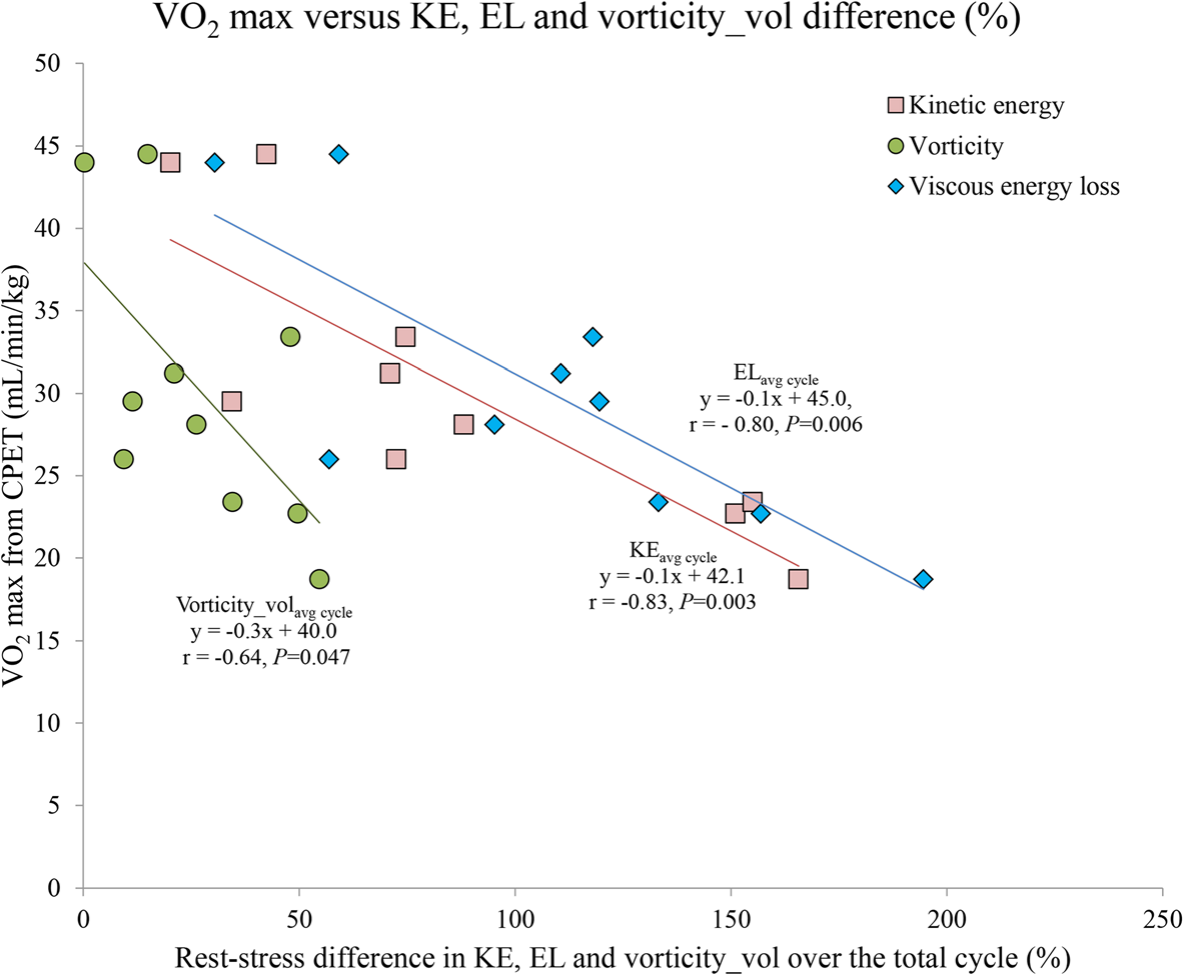
Scatter plot showing the relation between maximal oxygen uptake (VO_2_ max) from cardiopulmonary exercise tests (CPET) and the relative difference in intraventricular kinetic energy (KE), viscous energy loss (EL) and vorticity (vorticity_vol) over the total cardiac cycle between rest and stress

Figure 3 shows the scatter plot between VO_2_ max from CPET and the relative rest-stress difference in KE, EL and vorticity. This plot shows a significant good inverse correlation between VO_2_ max from CPET and relative KE_avg cycle_ rest-stress difference (*r* = − 0.83, *P* = 0.003). Furthermore, there is a significant good inverse correlation between VO_2_ max from CPET and relative EL_avg cycle_ rest-stress difference (*r* = − 0.80, *P* = 0.006). Also, VO_2_ max from CPET and relative vorticity_vol_avg cycle_ rest-stress difference show a significant moderate inverse correlation (*r* = − 0.64, *P* = 0.047). In the two patients with normal VO_2_ max [19] the relative rest-stress difference in vorticity_vol was 0.3–15%, the relative rest-stress difference in KE 20–42% and in EL 30–59%. The patient with the lowest VO_2_ max showed a 55% increase in vorticity_vol, but a 166% increase in KE and a 195% increase in EL during stress. Overall, decreased VO_2_ max is related to increases in vorticity_vol, KE and EL rest-stress differences, though both relative rest-stress differences in KE and EL showed a three-fold stronger increase than relative difference in vorticity_vol.

## Discussion

In the current study, the effect of pharmacologic stress on intraventricular KE, EL and vorticity, derived from 4D flow CMR was evaluated in Fontan patients and the relation was tested between stress response on these markers and exercise capacity, measured by VO_2_ max from cardiopulmonary exercise testing. Main findings of the study are: 1) intraventricular KE, EL and vorticity_vol increased during pharmacologic stress; 2) the increase in KE and EL under stress was 3–4 times higher than the increase in vorticity_vol; 3) VO_2_ max from CPET showed a significant negative correlation with the relative rest-stress differences in KE, EL and vorticity_vol. 4) at identical VO_2_ max value, KE and EL showed a three-fold higher increase between rest and stress compared to relative rest-stress difference in vorticity_vol. In the normal left ventricle at rest, blood flow follows the path that is most efficient for optimal ejection of flow in the systemic circulation. The shape of the healthy left ventricle and normal mitral valve contributes to the formation of a recirculating flow pattern during diastole, forming a confined region of vortical flow that conserves momentum by storing KE, while during systole the flow follows a rather semi-circular path from the mitral valve towards the left ventricular outflow tract [14, 15]. During stress the complex dynamic flow patterns in the heart might change in order to achieve redirection of momentum through the curved paths of flow [26]. An in vivo study in the hearts of healthy pigs [27] showed increased 4D flow CMR-derived trans-mitral diastolic inflow velocity and unfavorable conditions for diastolic vortex development under dobutamine stress. However, the effect of pharmacologic stress on 4D flow CMR-derived intraventricular flow patterns and KE, EL and vorticity in the human healthy left ventricle, has not been published.

Fontan patients have various structural ventricular and valvular anomalies. Moreover, the ventricle that sustains the systemic circulation can be of left ventricular or right ventricular morphology. Furthermore, in some patients, diastolic inflow occurs in the same ventricle as from which blood is ejected during systole (i.e., as is the case in a patient with tricuspid atresia with a normal ventriculo-arterial connection) while in other patients, inflowing blood has to pass through a ventricular septal defect first to reach the aorta for ejection (i.e., as is the case in patients with double inlet left ventricle with the aorta arising from a hypoplastic right ventricle). The abnormal intracardiac anatomy in Fontan patients causes differences in intraventricular flow patterns, which can be assessed and studied by 4D flow CMR [17]. In a similar way, altered flow patterns have been visualized and quantified in patients with a corrected atrioventricular septal defect [28] in which altered vortex formation was associated with increased EL obtained by 4D flow CMR [21]. Furthermore, in a patient with a Fontan circulation with a complete atrioventricular septal defect and a double outlet right ventricle with pulmonary stenosis, abnormal intracardiac structures have been linked to regions of vortex formation and increased EL [16]. In Fontan patients, decreased diastolic KE was reported versus healthy controls [18], as well as increased EL [22]. However, all these published studies focused on patients at rest and the response in intracardiac 4D flow CMR-derived hemodynamic parameters such as KE, EL and vorticity during stress has not been evaluated earlier.

Currently 4D flow CMR is challenging during physical exercise due to the incompatibility of the long scans times needed for 4D flow imaging and the movements inherent to physical exercise within the CMR scanner. Our study shows that pharmacologic (dobutamine-induced) stress results in a significant increase in intracardiac KE, EL and vorticity_vol from 4D flow CMR. The percentage increase was different between all individual subjects (Fig. 2, Table 3 in Appendix 2), which is dependent on ventricular size, the systemic ventricle, and could also be dependent on whether the patient has an extracardiac conduit or a lateral tunnel. The sample size is too small to permit more detailed analysis but it is clear that the only patient with a lateral tunnel (subject 5) has a different response to stress compared to a different patient with similar underlying anatomy but with a extracardiac conduit (subject 6). We could speculate that this may be explained by preload dependency i.e. it could mean that the lateral tunnel (which is an intra-atrial tunnel) inhibits preload under stress.

Notably, in the current study the dobutamine stress-induced increase in KE and EL is 3–4 times higher than the increase in vorticity_vol (KE_avg cycle_ increase 88%; EL_avg cycle_ increase 108%, vorticity_vol_avg cycle_ increase 27%). The difference in the increase in KE and EL compared to vorticity_vol could partly be explained by the abnormal underlying anatomy of the Fontan patients. Increased KE results in higher EL, which is possibly enhanced by more flow-structure interaction due to abnormal anatomy. However, the increase in vorticity_vol is not in the same order of magnitude. Vorticity in the ventricular flow might have a favorable effect (i.e.*,* conservation of momentum by KE storage and redirection of flow during diastole which takes place in a confined region of vortical flow distal to the mitral valve) [14, 15]. However, vorticity may also have an adverse effect on the intracardiac blood flow when optimal flow redirection may be perturbed by flow separation and vortex shedding distal to abnormal intracardiac structures in the flow field [29–31]. In this study, we are not able to discriminate between favorable and adverse effects of vorticity. In either case, the KE contributes to vortex formation but this study showed that stress results in a higher increase in KE than in vorticity. The relation between vortex formation and flow velocity (i.e. kinetic energy) has been studied before: Gharib et al. [29] described that the volume and circulation of a vortex ring that is formed from inflow through a circular nozzle into a large unconfined volume would both increase with increasing inflow velocity, but only up to a critical point where optimal vortex formation has been achieved. At this optimal point, both volume and vorticity of the main vortex will remain approximately constant. However, secondary vortices may start to appear that will contribute to the total summed vorticity. Furthermore, in our study the shape of the ventricle measured by the sphericity index and end-diastolic volume remained similar during rest and stress. This could support the hypothesis that the size of the vortical flow region during diastole is restricted in growth and unable to expand at the same rate as the KE increase during stress.

Survival after the Fontan procedure has increased drastically over the past decades [2, 3]. Still, patients with a Fontan circulation exhibit diminished exercise capacity, related to worse functional health status [4–6]. Several factors have been identified that play a role in the reduced exercise capacity in these patients [7–11]. However, the only previous study that related exercise capacity to EL was aimed at the TCPC of Fontan patients [10] and not at intraventricular flow dynamics. In the current study, an increase in the relative rest-stress difference in KE, EL and vorticity_vol was inversely correlated with VO_2_ max, though notably relative rest-stress differences in KE and EL showed a three-fold stronger increase than relative difference in vorticity_vol. This is in line with our previous speculation that, in patients with lower exercise capacity, storage capacity of KE inside the confined vortical flow region is limited and therefore, does not proportionally follow the increase in total KE. Furthermore, abnormal intraventricular anatomy could play a role in this relation as well. Nevertheless, our findings may provide evidence that intracardiac hemodynamics and energetics play a role in exercise capacity in Fontan patients.

Our results showed no association between the difference in HR and the difference in KE, EL or vorticity. However, the HR increase during the low-dose dobutamine stress protocol we used is limited to a 30% increase. From this study, we cannot determine whether this relation would be similar when HR increase is higher. Also, we showed that SV difference is directly related to vorticity difference, but not to the difference in KE or EL.

In the current study we evaluated intracardiac 4D flow hemodynamic parameters in a rest –stress setting and associated the results with an exercise parameter (VO_2_ max). In Fontan patients, there is a delicate interplay between preload and afterload and efficiency of the TCPC and aortic outflow is also of great importance. This study is a first step in the evaluation of the intricate hemodynamic status of the Fontan patient in exercise. Future studies should also relate TCPC hemodynamics during stress with intracardiac hemodynamics during stress.

### Limitations

This study has a number of limitations. One limitation is the lack of a control group in order to compare these results in Fontan patients to a normal range. However, studying the effect of pharmacological (dobutamine) stress on 4D flow CMR-derived intraventricular flow patterns and KE, EL and vorticity in the healthy left ventricle in children requires a strict and thorough ethical consideration and the current study was not eligible for such test. Another limitation of the current study is the small sample size. However, our results are still highly statistically significant. Because of the small sample size, we could not evaluate individual differences between patients with separate underlying anatomies. Furthermore, we did not include an evaluation of turbulent kinetic energy, which could be relevant in Fontan patients, as turbulent flow may be present due to the structural intraventricular abnormalities. Additionally, 4D flow CMR is a time-resolved imaging modality that only represents time-averaged blood flow acquired over multiple heart cycles. Consequently, this limits the evaluation to coherent large-scale (within 4D flow CMR resolution) vortical flow only, while incoherent unstable or small-scale flow, especially during a stress-induced increase in cardiac workload, could not be measured, although the effect may not be negligible. We did not evaluate any changes in afterload induced by stress and therefore the influence of changes in afterload on ventricular energetics, which could be subject for further research. Lastly, in this study the 4D flow CMR results under pharmacologic stress were compared to VO_2_max during exercise stress. There could be a potential confounding effects of 4D flow CMR measurements performed while supine versus exercise measurements performed upright [32], especially in Fontan patients [33]. It would have been theoretically ideal to perform the exercise testing with a supine ergometer to minimize this confounder, however this was not possible in our study protocol. Further studies should evaluate this effect.

### Clinical perspectives

This study provides evidence that intracardiac vortical flow and energetics play a role in exercise capacity in Fontan patients. Although still speculative, these results could potentially influence patient follow-up and surgical planning. Insight in hemodynamic parameters during stress may provide a quantitative index that could help better understand how the mechanism of which underlying pathophysiology effects the response to stress and the influence on exercise capacity. However, larger future studies are needed to confirm the results of this pilot study.

## Conclusions

In conclusion, intraventricular kinetic energy, viscous energy loss and vorticity values derived from 4D flow CMR increased during pharmacologic stress, with a three to four times higher increase in KE and EL compared to the vorticity increase. The increase in KE, EL and vorticity showed a negative correlation with exercise capacity measured by VO_2_ max from cardiopulmonary exercise testing. At identical VO_2_ max value, both relative rest-stress differences in KE and EL showed a three-fold stronger increase than the relative differences in vorticity.

## Data Availability

The datasets used and/or analysed during the current study are available from the corresponding author on reasonable request.

## Appendix 1

### CMR acquisition information

A complete CMR scan including whole-heart 4D flow CMR was obtained on a 3 T scanner (Ingenia, Philips Healthcare, Best, the Netherlands) with maximal amplitude of 45 mT/m for each axis and slew rate of 200 T/m/s. A combination of FlexCoverage Posterior coil in the table top with a dStream Torso coil, providing up to 32 coil elements was used for signal reception. Velocity-encoding of 150 cm/s in all three directions was used in a standard four-point encoding scheme, spatial resolution 3.0 × 3.0 × 3.0 mm^3^ or better, flip angle 10°, echo time (TE) 3.7 ms, repetition time (TR) 7.7–10 ms, true temporal resolution 30–40 ms, sensitivity encoding factor 2 in anterior-posterior direction and echo planar imaging readout with a factor 5. Concomitant gradient correction and phase offset correction was performed using standard available scanner software. Typical acquisition time of the whole-heart 4D flow CMR scan was approximately 8 min. Cine two-dimensional left 2-chamber, 4-chamber, coronal and sagittal aorta views and transversal images were acquired, using balanced steady-state free-precession sequences with TE/TR 1.5/3.0, 350 mm field-of-view, 45° flip angle, acquisition resolution 1.9 × 2.0 × 8.0 mm^3^. Retrospective gating was used with 30 phases reconstructed to represent one cardiac cycle. Free breathing was allowed without using motion suppression; three signal averages were taken to minimize effects of breathing motion.

## Appendix 2

**Table 3:** Subject specific parameters

Subject specific parameters								
Subject	Underlying anatomy	Ventricular morphology	Type of Fontan	VO2max^a^	Rest-stress diff EDV (%)	Rest-stress diff KE_avg cycle_ (%)	Rest-stress diff EL_avg cycle_ (%)	Rest-stress diff Vorticity_vol_avg cycle_ (%)
1	Unbalanced AVSD, hypoplastic LV	Biventricular	Extracardiac	33.4	13.07	74.64	58.17	47.98
2	TGA,VSD, PS, hypoplastic LV	Biventricular	Extracardiac	22.7	.71	151.04	93.35	49.66
3	ccTGA, VSD, PS, straddling TV	Biventricular	Extracardiac	29.5	−12.97	34.45	77.63	11.44
4	PA	Left	Extracardiac	18.7	− 2.54	165.67	39.92	54.71
5	TA	Left	Lateral tunnel	26.0	−16.70	72.51	8.07	9.40
6	TA	Left	Extracardiac	44.0	−8.00	20.18	−3.54	.31
7	PA	Left	Extracardiac	23.4	−9.23	155.04	80.78	34.58
8	DILV, with aorta from RV	Left	Extracardiac	31.2	−10.15	71.06	40.02	21.10
9	HLHS	Right	Extracardiac	28.1	−2.88	88.17	71.19	26.23
10	TGA,VSD, PS, hypoplastic LV	Right	Extracardiac	44.5	−1.35	42.44	7.91	14.96

^a^from cardiopulmonary exercise testing

*EDV* end diastolic volume, *VO2 max* maximal oxygen uptake, *AVSD* atrioventricular septal defect, *LV* left ventricle, *TGA* transposition of the great arteries, *VSD* ventricular septal defect, *PS* pulmonary stenosis, *TV* tricuspid valve, *PA* pulmonary atresia, *TA* tricuspid atresia, *DILV* double inlet right ventricle, *RV* right ventricle, *HLHS* hypoplastic left heart syndrome

## Abbreviations

4D: Four-dimensional
CI: Confidence interval
CMR: Cardiovascular magnetic resonance
CO: Cardiac output
CPET: Cardiopulmonary exercise testing
EDV: End-diastolic volume
EF: Ejection fraction
EL: Viscous energy loss
ESV: End-systolic volume
HR: Heart rate
IQR: Interquartile range
KE: Kinetic energy
LV: Left ventricle/left ventricular
SV: Stroke volume
TCPC: Total cavopulmonary connection
VO_2_ max: Maximum oxygen uptake
vorticity_vol: Vorticity integral over the ventricular volume
